# Disrupting the Btk Pathway Suppresses COPD-Like Lung Alterations in Atherosclerosis Prone ApoE^−/−^ Mice Following Regular Exposure to Cigarette Smoke

**DOI:** 10.3390/ijms19020343

**Published:** 2018-01-24

**Authors:** Jon M. Florence, Agnieszka Krupa, Laela M. Booshehri, Adrian L. Gajewski, Anna K. Kurdowska

**Affiliations:** 1 Department of Cellular and Molecular Biology, University of Texas Health Science Center, Tyler, TX 75708, USA; jon.florence@uthct.edu (J.M.F); agakrupa@yahoo.com (A.K.); laela.booshehri@gmail.com (L.M.B.); gajewski@biol.uni.lodz.pl (A.L.G.); 2 Laboratory of Gastroimmunology, Department of Immunology and Infectious Biology, Faculty of Biology and Environmental Protection, University of Lodz, 90-237 Lodz, Poland

**Keywords:** chronic lung inflammation, emphysema, second hand smoke, Bruton’s tyrosine kinase, matrix metalloproteinase-9

## Abstract

Chronic obstructive pulmonary disease (COPD) is associated with severe chronic inflammation that promotes irreversible tissue destruction. Moreover, the most broadly accepted cause of COPD is exposure to cigarette smoke. There is no effective cure and significantly, the mechanism behind the development and progression of this disease remains unknown. Our laboratory has demonstrated that Bruton’s tyrosine kinase (Btk) is a critical regulator of pro-inflammatory processes in the lungs and that Btk controls expression of matrix metalloproteinase-9 (MMP-9) in the alveolar compartment. For this study apolipoprotein E null (ApoE^−/−^) mice were exposed to SHS to facilitate study in a COPD/atherosclerosis comorbidity model. We applied two types of treatments, animals received either a pharmacological inhibitor of Btk or MMP-9 specific siRNA to minimize MMP-9 expression in endothelial cells or neutrophils. We have shown that these treatments had a protective effect in the lung. We have noted a decrease in alveolar changes related to SHS induced inflammation in treated animals. In summary, we are presenting a novel concept in the field of COPD, i.e., that Btk may be a new drug target for this disease. Moreover, cell specific targeting of MMP-9 may also benefit patients affected by this disease.

## 1. Background

Second hand smoke (SHS) can illicit damage to lung tissue by altering signaling pathways that regulate both inflammatory responses and repair processes in the alveolar compartment. In fact, the induction of abnormal inflammation by SHS is a well-recognized underlying cause of pathogenic features characteristic of chronic obstructive pulmonary disease (COPD) [1]. A substantially enhanced inflammatory immune response in the airways and lungs is a hallmark of COPD. Natural history of the disease classically begins with inflammatory changes in the larger airways (chronic bronchitis). Remodeling and narrowing of the small airways and parenchymal tissue destruction with airspace enlargement (emphysema) are also well-recognized features of COPD [1,2].

Development of novel therapies for COPD would not be possible without animal models that adequately reflect pathophysiology of this disease. Animal models utilizing cigarette smoke exposure display the characteristic features of human COPD including accumulation of inflammatory cells, small airway fibrosis/remodeling, mucus hyper-secretion, lung dysfunction and development of emphysema [3]. In mouse models of COPD, chronic exposure of mice to cigarette smoke triggers typical pathological hallmarks of the disorder, such as pulmonary inflammation, airway remodeling and airspace enlargement caused by the destruction of alveolar walls [3,4,5,6,7,8,9,10].

Novel studies from our laboratory have shown that activation of Bruton’s tyrosine kinase (Btk) was significantly increased in lungs during severe inflammation [11,12]. Btk belongs to a family of Tec kinases, non-receptor intracellular tyrosine kinases. Tec kinases typically reside in an inactive form in the cytoplasm, and are translocated to the membrane fraction upon cell stimulation where they initiate downstream signaling cascades [11,13]. We have also noted that Btk may regulate expression of matrix metalloproteinase-9 (MMP-9) in the lung [11,12]. Matrix metalloproteinases (MMPs) are proteolytic enzymes capable of degrading matrix components. This process is beneficial in normal physiological states, but can be harmful in pathological conditions. Significantly, increased levels of MMP-9 have been detected in the lungs of smokers with COPD [14] as well as smoke exposed mice [5,10,15].

It should be stressed that exposure of mice to SHS remains the best animal system for defining, testing, and evaluating novel drug targets for COPD [3]. The model of apolipoprotein E deficient (ApoE^−/−^) mice that we employed in the study mimics systemic co-morbidities of COPD and accurately reflects multiple aspects of the corresponding clinical disease [4,5,6,7,15]. We have applied two types of treatments to ApoE^−/−^ mice exposed to SHS. The animals received either a pharmacological inhibitor of Btk or MMP-9 specific siRNA to minimize MMP-9 expression in endothelial cells or neutrophils. We previously reported on the impact of these treatments with respect to the atherosclerotic aspect of this model [16]. Here we show that all treatments resulted in reduction of pulmonary changes related to COPD progression. Targeting Btk or cell specific expression of MMP-9 may be prospective treatments for COPD patients.

## 2. Results

### 2.1. Exposure to SHS Induces Alveolar Destruction and Airspace Enlargement in Lungs of ApoE^−/−^ Mice

**Figure 1:**
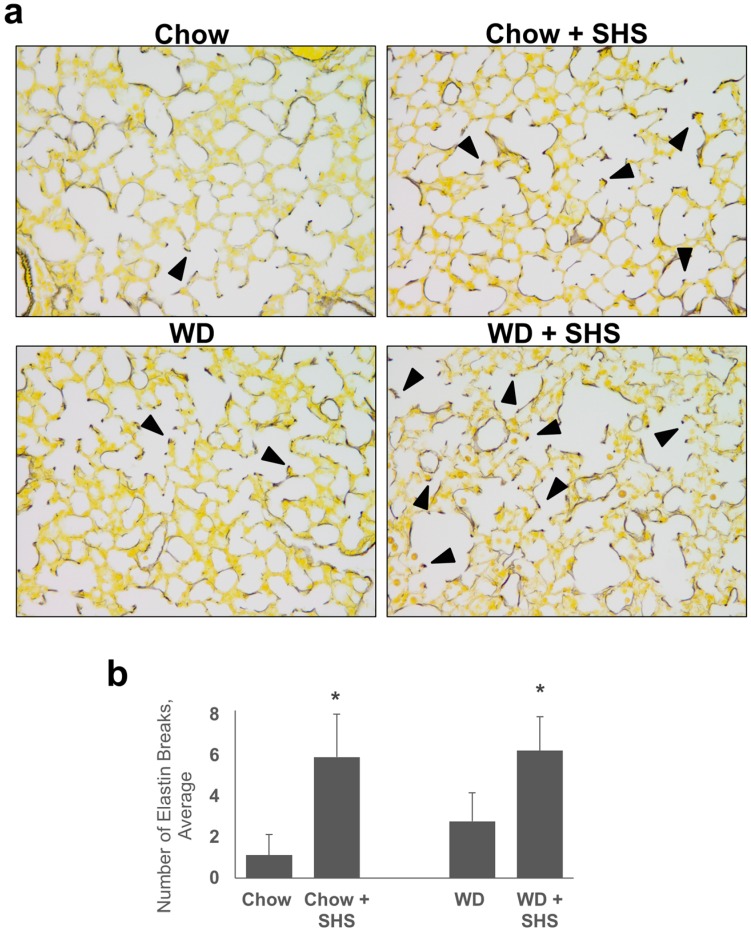
Hart’s Elastin stained lung tissue sections from ApoE^−/−^ mice. ApoE^−/−^ mice were fed rodent chow without (Chow) or with second hand smoke exposure (Chow + SHS). Other groups were fed Western Diet without (WD) or with smoke exposure (WD + SHS). (**a**) Representative images are shown. Black arrows point out strand breaks in alveolar walls, 20x objective used. Destruction of alveolar walls was assessed by counting numbers of breaks in elastin fibers (**b**). Groups exposed to SHS scored significantly higher, the Kruskal-Wallis ANOVA on ranks was used to assess statistical significance between groups (*p* < 0.001) followed by post hoc testing with Dunn’s multiple comparison test for groups of interest (*p* < 0.05), four to six mice per group were analyzed, * Significant difference detected.

Hart’s Elastin stain was used to assess alveolar wall destruction in lungs of ApoE^−/−^ mice on standard rodent chow or “Western Diet” (WD) fed mice with or without SHS exposure. Evaluation of alveolar wall condition was based on observation of airspace elastin, its appearance and continuance. In normal mice airspace elastin appears as thin continuous strands which outline alveolar walls [17,18]. As shown in Figure 1a, alveolar destruction in the SHS exposed animals is visible as a loss of elastic round, intact alveoli. Moreover appearance of elastin nodules is a consequence of alveolar destruction, and result from recoil of severed elastin strands upon loss of tension [19,20]. Black arrows denote possible breaks in elastic fibers in lungs of mice exposed to SHS. Destruction of alveolar walls was assessed by counting numbers of breaks in elastic fibers and the numbers were significantly higher in smoke exposed animals (Figure 1b). 

**Figure 2:**
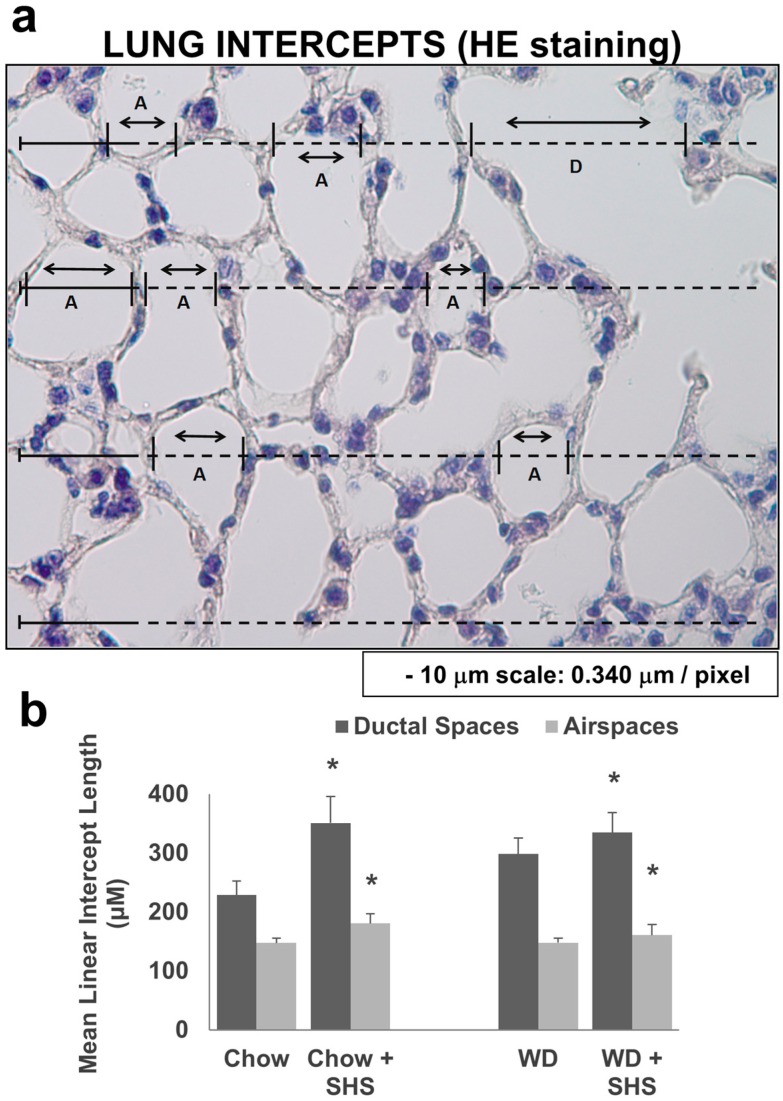
Morphometric analysis of lung sections from ApoE^−/−^ mice fed rodent chow without (Chow) or with second hand smoke exposure (Chow + SHS) or fed Western Diet without (WD) or with smoke exposure (WD + SHS). Representative image of hematoxylin and eosin stained lung tissue section from ApoE^−/−^ mice (Air spaces—A and Ductal spaces—D) (**a**). The MLI calculated using hematoxylin and eosin stained lung sections (**b**). Groups exposed to SHS scored significantly higher. For airspace group scores the Kruskal–Wallis ANOVA on ranks was used to assess statistical significance (*p* < 0.001) followed by post hoc testing with Dunn’s multiple comparison test for Chow ± SHS groups (*p* < 0.05). As Dunn’s test did not give a p value for the pairwise comparison of WD ± SHS groups, the non-parametric Mann-Whitney test with Bonferroni correction for multiple comparisons was used instead (*p* = 0.016). For ductal space group scores one way ANOVA was used (*p* < 0.001) followed by post hoc Fisher’s least significant difference tests. Chow ± SHS (*p* < 0.001), WD ± SHS (*p* = 0.004), three to five mice per group were evaluated. * Significant difference detected.

To study the differences in airspace enlargement in lungs of ApoE^−/−^ mice with or without cigarette smoke exposure we calculated the mean linear intercept length (MLI) [21] using Hematoxylin and Eosin stained lung tissue sections. As shown in Figure 2, the MLI was significantly higher in smoke exposed mice fed regular diet (Figure 2B) as well as in mice fed WD and exposed to smoke relative to non-smoking groups (Figure 2B).

### 2.2. Exposure to SHS Triggers an Increase in Airway Wall Collagen in Lungs of ApoE^−/−^ Mice

**Figure 3:**
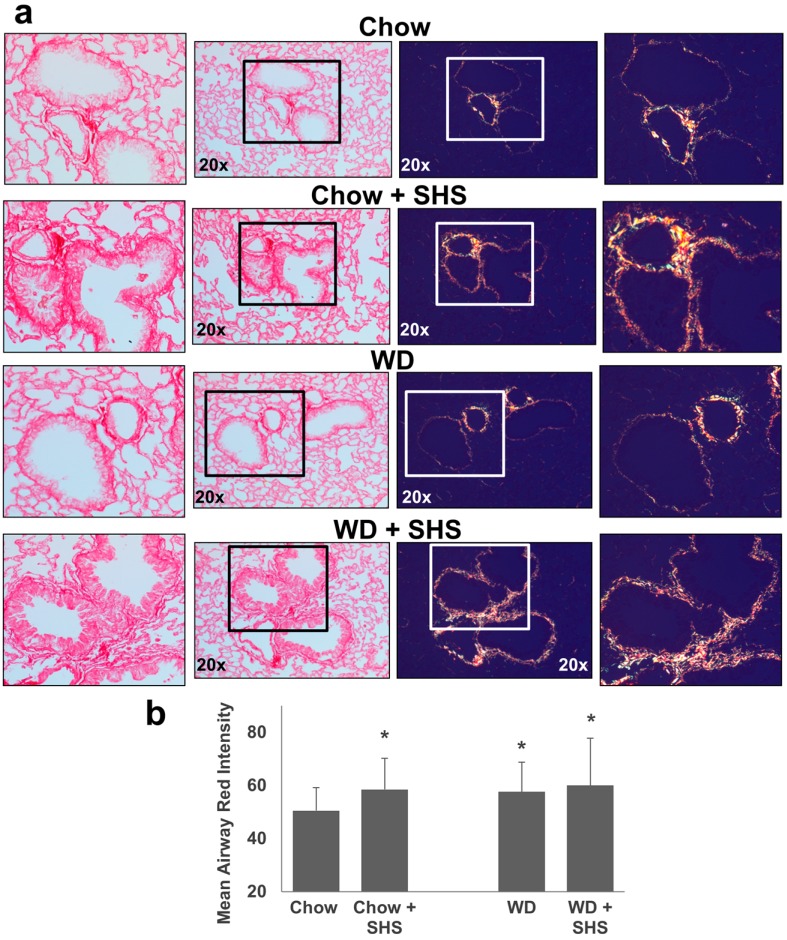
Collagen deposition in lungs of ApoE^−/−^ mice fed rodent chow without (Chow) or with second hand smoke exposure (Chow + SHS) or fed Western Diet without (WD) or with smoke exposure (WD + SHS). Picro-sirius red stained lung tissue sections were viewed under regular light (left panels) or polarized light (right panels). Representative images are shown (**a**). Differences in intensity of red-orange birefringence from Picro-sirius red stained airways were evaluated using ImageJ software. Results were expressed as mean red component intensities (**b**). Groups exposed to SHS and/or WD scored significantly higher than the chow fed, air only group (*p* < 0.05). Kruskal-Wallis ANOVA on ranks was used to assess statistical significance between groups (*p* < 0.001) followed by post hoc testing with Dunn’s multiple comparison test for groups of interest, four to five mice per group were evaluated. * Significant difference detected.

Picro-Sirius Red stained lung sections were examined under plane polarized light to visualize collagen content of the airway walls. This method is known to have higher specificity for collagen as well as allowing differentiation between thick and fine fibers missed by traditional trichrome methods [22]. When viewed with polarized light the hue of collagen fibers is indicative of collagen fiber thickness with very fine fibers appearing green while thick fibers produce a yellow to orange/red birefringence with respect to increasing thickness [22]. As shown in Figure 3 the layer of airway collagen is wider and comprised much more of thick fibers in smoke exposed and/or western diet fed mice relative to non-smoking chow fed controls as indicated by the shift in birefringence (increased orange/red color). Statistical analysis is presented in Figure 3b. 

### 2.3. Treatments with a Pharmacological Inhibitor of Btk or MMP-9 Specific siRNA Targeting Either Endothelial Cells or Neutrophils Causes a Decrease in Alveolar Changes Related to COPD Progression

We subsequently applied long term treatments to ApoE^−/−^ mice fed WD and exposed to SHS. As these mice had increased lung MMP-9 (Figure S1), treatments were designed to directly or indirectly reduce lung MMP-9 levels (Figure S2). The animals received either a pharmacologic inhibitor of Btk (PCI-32765) or MMP-9 directed siRNA designed to minimize cell specific MMP-9 expression in endothelial cells or neutrophils. These treatments were protective in ApoE^−/−^ mice fed WD and exposed to SHS. 

**Figure 4:**
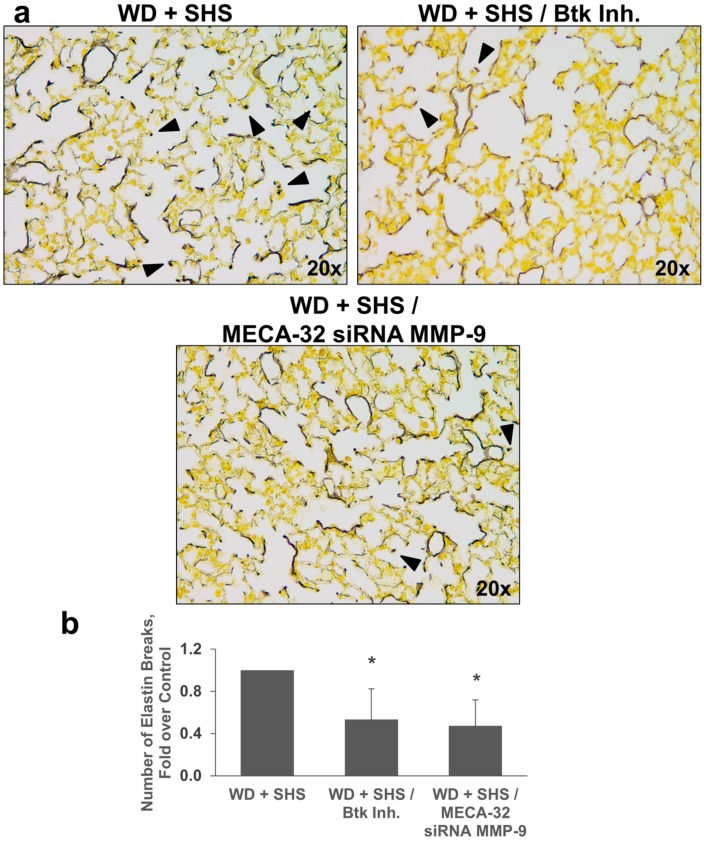
Analysis of alveolar elastin breaks in lungs of ApoE^−/−^ mice fed WD and exposed to SHS only (WD + SHS), with Btk inhibitor treatment (WD + SHS/Btk Inh.), or treated with endothelial cell targeted siRNA for MMP-9 (WD + SHS/MECA-32 siRNA MMP-9). Representative images are shown. Black arrows point out strand breaks in alveolar walls (**a**). Destruction of alveolar walls was assessed by counting numbers of breaks in elastin fibers (**b**). Groups receiving treatment scored significantly lower than the control group (*p* < 0.05). Kruskal–Wallis ANOVA on ranks was used to assess statistical significance between groups (*p* < 0.001) followed by post hoc testing with Dunn’s multiple comparison test for groups of interest, four to six mice per group were evaluated. * Significant difference detected.

**Figure 5:**
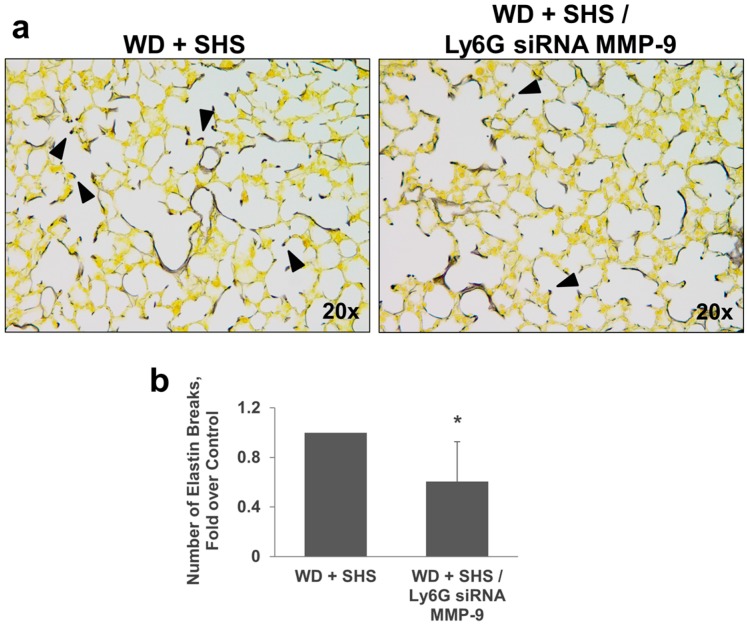
Analysis of alveolar elastin breaks in lungs of ApoE^−/−^ mice fed WD and exposed to SHS only (WD + SHS) or treated with neutrophil targeted siRNA for MMP-9 (WD + SHS/Ly6G siRNA MMP-9). Black arrows point out strand breaks in alveolar walls (**a**). Destruction of alveolar walls was assessed by counting numbers of breaks in elastin fibers (**b**). The treatment group scored significantly lower than the control groups (*p* < 0.001). The non-parametric Mann–Whitney test was used to assess statistical significance between groups, four to five mice per group were analyzed. * Significant difference detected.

Hart’s Elastin stain was used to analyze airway destruction/strand breaks in alveolar walls of ApoE^−/−^ mice exposed to cigarette smoke and treated with Btk inhibitor, or MMP-9 siRNA targeting endothelial cells and neutrophils. As shown in Figure 4, fewer elastin breaks were observed in lungs of mice treated with Btk inhibitor and with MMP-9 siRNA targeting endothelial cells (Figure 4) or neutrophils (Figure 5). 

**Figure 6:**
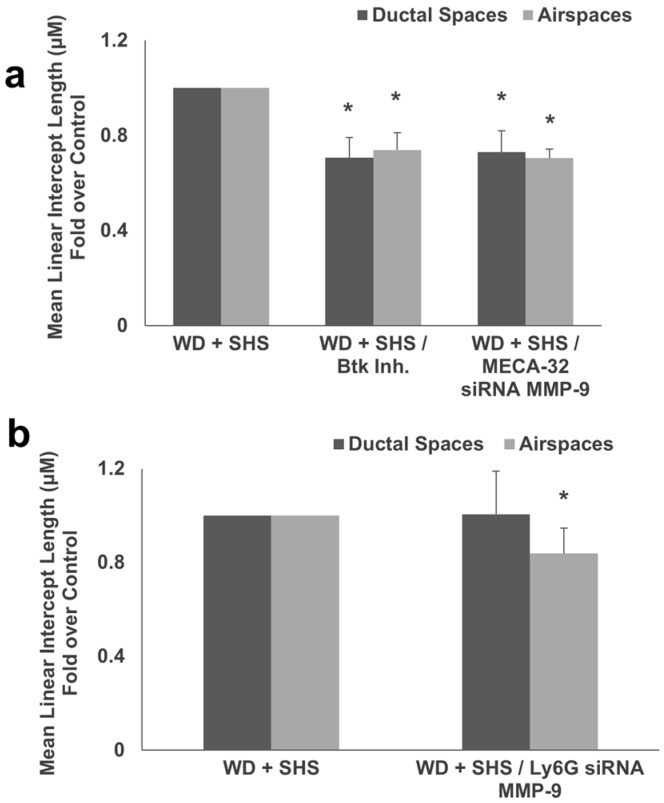
(**a**) Morphometric analysis of lungs from ApoE^−/−^ mice fed WD and exposed to SHS only (WD + SHS), with Btk inhibitor treatment (WD + SHS/Btk Inh.), or treated with endothelial cell targeted siRNA for MMP-9 (WD + SHS/MECA-32 siRNA MMP-9) showed reduced scores in treatment groups (*p* < 0.05). Kruskal-Wallis ANOVA on ranks was used to assess statistical significance between groups (*p* < 0.001) followed by post hoc testing with Dunn’s multiple comparison test for groups of interest, four to six animals per group were evaluated, with two groups of control animals combined. (**b**) Analysis of MLI in lungs of ApoE^−/−^ mice fed WD and exposed to SHS or fed WD and exposed to SHS plus treatment with neutrophil targeted siRNA for MMP-9 (WD + SHS/Ly6G siRNA MMP-9) showed reduced scores in the treatment group (*p* > 0.001). Student’s *t*-test was used to assess statistical significance, four mice per group were analyzed. * Significant difference detected.

Hematoxylin and eosin staining was used for morphometric analysis of airspace enlargement and to calculate MLI length which was significantly lower in mice that received the Btk inhibitor and MMP-9 siRNA targeting endothelial cells (Figure 6a), as well as in mice treated with MMP-9 directed siRNA designed to minimize cell specific MMP-9 expression in neutrophils (Figure 6b). 

**Figure 7:**
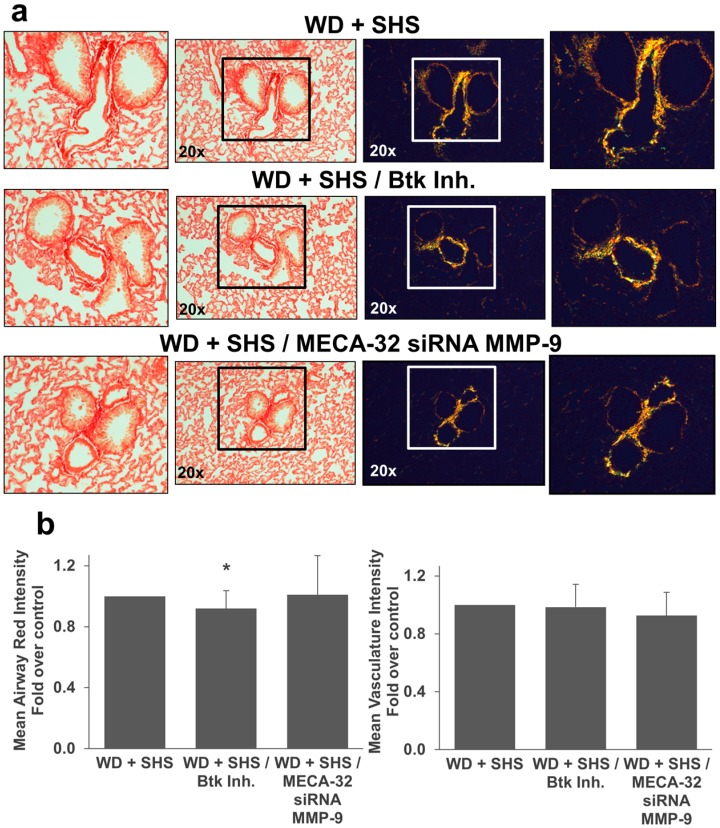
Collagen deposition in lungs of ApoE^−/−^ mice fed WD and exposed to SHS only (WD + SHS), with Btk inhibitor treatment (WD + SHS/Btk Inh.), or treated with endothelial cell targeted siRNA for MMP-9 (WD + SHS/MECA32 siRNA MMP-9). Picro-sirius red stained lung sections from ApoE^−/−^ mice were viewed under regular light (left panels) or polarized light (right panels). Representative images are shown (**a**). Differences in hue of Picro-sirius red stained airways were evaluated using ImageJ software. Results are expressed as mean red component intensities (airway) or mean total intensity (vasculature) (**b**). Btk inhibition or EC targeted MMP-9 specific siRNA impacted collagen deposition in the airways or lung vasculature respectively. Kruskal-Wallis ANOVA on ranks was used to assess statistical significance in red intensity between groups (*p* < 0.081). As Dunn’s multiple comparison test was not possible we assessed the reduction observed in the Btk inhibitor treated group using the non-parametric Mann-Whitney test with Bonferroni correction for multiple comparisons (*p* = 0.019). For mean vascular intensity one way ANOVA was used to assess differences between groups (*p* = 0.227) and as Fishers least significant difference test was not possible, we assessed the reduction observed in the MECA-32 siRNA MMP-9 treated group using Student’s *t*-test with Bonferroni correction for multiple comparison (*p* = 0.097), four to six animals per group were evaluated, with two groups of control animals combined. * Significant difference detected.

**Figure 8:**
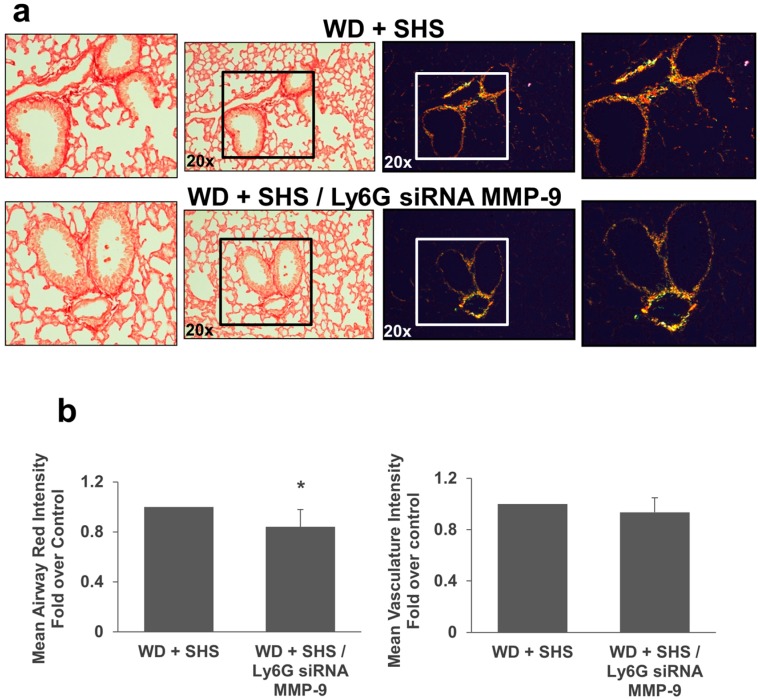
Collagen deposition in lungs of ApoE^−/−^ mice fed WD and exposed to SHS only (WD + SHS), or treated with PMN targeted siRNA for MMP-9 (WD + SHS/Ly6G siRNA MMP-9). Picro-sirius red stained sections from ApoE^−/−^ mice were viewed under regular light (left panels) or polarized light (right panels). Representative images are shown (**a**). Differences in hue of Picro-sirius red stained airways were evaluated using ImageJ software. Results are expressed as mean red component intensities (airway) or mean total intensity (vasculature) (**b**). PMN targeted MMP-9 specific siRNA impacted collagen deposition. The non-parametric Mann-Whitney test (airway, *p* > 0.001) or Student’s *t*-test (vasculature, *p* = 0.098) were used to assess statistical significance between groups as indicated by group normality, five mice per group were evaluated. * Significant difference detected.

Moreover, we employed Picro-Sirius Red staining to analyze airway collagen deposition in lungs of ApoE^−/−^ mice exposed to smoke and treated with Btk inhibitor or MMP-9 siRNA. As shown in Figure 7 there was a decrease in collagen deposition and less thick collagen fibers in treated animals relative to control mice. Collagen deposition in airways was significantly decreased in mice treated with the Btk inhibitor (Figure 7b). The difference in lung vasculature collagen content did not reach statistical significance for treated mice compared to untreated animals although vascular collagen content was substantially lower in mice that received endothelial cell targeted MMP-9 specific siRNA (Figure 7b). We also noted a decrease in airway collagen deposition in mice that received neutrophil targeted MMP-9 directed siRNA (Figure 8a,b) and again vascular collagen content was substantially lower in mice that received neutrophil targeted MMP-9 specific siRNA but this difference did not reach statistical significance.

**Figure 9:**
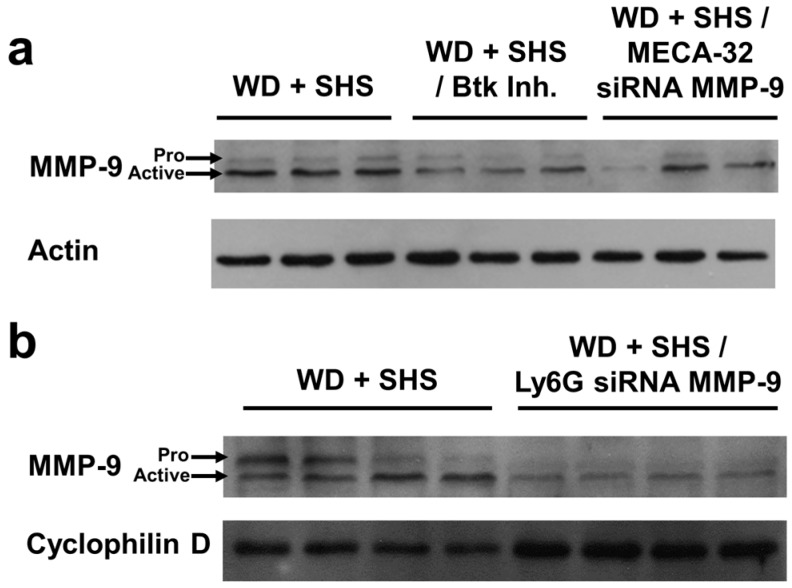
Levels of MMP-9 in lung homogenates (**a**). Western blot from ApoE^−/−^ mice fed WD and exposed to SHS only (WD + SHS), with Btk inhibitor treatment (WD + SHS/Btk Inh.), or treated with endothelial cell targeted siRNA for MMP-9 (WD + SHS/MECA-32 siRNA MMP-9). Three mice per group were analyzed (**b**). Western Blot from ApoE^−/−^ mice fed WD and exposed to SHS only (WD + SHS) or treated with neutrophil targeted siRNA (WD + SHS/Ly6G siRNA MMP-9). Four animals per group were analyzed.

Finally, we tested the level of MMP-9 in lung homogenates of ApoE^−/−^ mice exposed to smoke and treated with Btk inhibitor and MMP-9 siRNA targeting endothelial cells and neutrophils. Figure 9a and supplemental Figure S3 shows that the level of MMP-9 is reduced in mice that received the Btk inhibitor as well as animals that were treated with MMP-9 directed siRNA targeted to endothelial cells. Reduced lung MMP-9 in animals treated with MMP-9 directed siRNA targeting neutrophils is shown in Figure 9b.

## 3. Discussion

Emphysema/COPD, a smoking-related complex inflammatory airway disease, is the third leading cause of death in the United States [1,3]. Persistent chronic inflammation associated with COPD eventually triggers irreversible tissue destruction [1]. Current treatments for COPD do not effectively inhibit chronic inflammation or reverse the pathology of disease, and at the same token do not successfully target the factors that initiate and drive the long-term progression of disease. Regrettably treatment options are very limited and mainly focus on improving quality of life [2]. This limitation is due to a lack of understanding for the mechanisms and mediators that drive the induction and progression of chronic inflammation, emphysema and altered lung function. It should be stressed that airflow limitation in COPD is not fully reversible. At present, casual interventions that can stop progression of this disease are not available [1]. There is a clear need for new therapies that can prevent the induction and progression of COPD [3]. 

Animal models remain essential for the development of novel therapies. Exposure of mice to cigarette smoke triggers characteristic features of human COPD including the accumulation of pro-inflammatory cells and mediators, small airway fibrosis/remodeling, mucus hypersecretion, lung dysfunction, and the development of emphysema. In addition, models that mimic systemic co-morbidities are equally important [3,4,5,6,7,8]. Moreover, existing treatments for COPD are mainly focused on symptom alleviation (especially dyspnea) and reduction in exacerbations [2,23]. A hallmark of COPD is an enhanced inflammatory immune response in the airway and the lung. Targeting this pathway is a logical approach to the treatment of COPD [2].

Vascular abnormalities are well known as comorbidities of COPD [24,25]. They include endothelial dysfunction, arterial stiffness and atherogenesis. Furthermore pulmonary vascular collagen deposition is observed in patients with mild COPD as well as smokers suggesting it too contributes to the web of comorbidities associated with COPD [26]. Similarly, cardiovascular problems go hand in hand with decline in lung function in smokers. In addition, recent studies indicate that vascular inflammation, endothelial dysfunction and oxidative modification of lipids may contribute to the pathogenesis of COPD [25,27,28]. Therefore, it comes as no surprise that abnormal morphology and a substantial decrease in lung function are found in ApoE^−/−^ mice which are susceptible to cardiovascular issues and are prone to atherosclerosis [4,5,6,7,29]. Exposure to cigarette smoke causes premature emphysema, abnormal lung inflammation, and airspace enlargement with altered mechanical properties in lungs of these mice [4,5,6,7]. Additionally, deposition of thick collagen fibers around airways in smoke exposed animals, a suspected source of increased airway stiffness and associated airway resistance, was observed as well as vascular deposition. These observations have also been reported in an elastase induced emphysema mouse model [20]. In the study we employed atherosclerosis prone apolipoprotein E deficient (ApoE^−/−^) mice as an animal model which mimics systemic co-morbidities of COPD and accurately reflects the corresponding clinical disease [4,5,6,7,15]. Our findings in this emerging comorbidity model revealed reduced alveolar changes associated with COPD progression in cigarette exposed ApoE^−/−^ mice treated with either Btk inhibitor, or siRNA directed to MMP-9. Previous studies have made direct comparisons of lung alterations in ApoE^−/−^ and WT mice are made in the context of smoke exposure and western diet/HFD respectively. In one such study relative to WT mice ApoE^−/−^ mice were shown to have similar if not greater lung macrophage and neutrophil numbers following SHS exposure, as well as higher levels of lipid peroxidation, chemokines (MCP-1 and KC), MMP-9 and MMP-12, greater mean linear intercept, and reduced eNOS activity. Additionally several of these factors were increased significantly in ApoE^−/−^ mice relative to WT mice without smoke exposure [5]. Another study showed ApoE^−/−^ mice fed a high fat/high cholesterol (western) diet for 12 weeks had significantly increased septal thickening and mean linear intercepts relative to WT mice fed western diets and ApoE^−/−^ mice fed a standard chow diet as well as increases in CD68 and TLR4 positive cells in lung tissue. Furthermore ApoE^−/−^ mice fed a western diet had significantly higher BALF TNF-α, IL-4, IL-6, IL-17, and IFN-γ compared to normal chow fed ApoE^−/−^ mice [29]. Additionally, recent reviews contain excellent discussions on the emergence of this model in CVD/COPD comorbidity studies [4,30]. 

Btk plays a critical role in the pathophysiology of inflammatory lung diseases [11,12]. Therefore, we hypothesized that it may also contribute to alveolar changes related to progression of COPD. Indeed, administration of a specific pharmacological inhibitor of Btk was protective in ApoE^−/−^ mice fed WD and exposed to SHS. Further, MMPs, and specifically MMP-9, have been implicated in pathogenesis of COPD [14]. Brajer et al. showed a correlation between higher concentrations of MMP-9 in serum and increased progression of systematic inflammation in COPD patients [23]. Significantly, recent studies from our laboratory indicate that Btk may regulate expression of MMP-9 in lungs [11,12]. In agreement with this observation, we have noted a decrease in MMP-9 levels in lungs of mice treated with a Btk inhibitor. Moreover, animals that received siRNA specific for MMP-9 targeted to endothelial cells or neutrophils through conjugation with F(ab’)_2_ fragments of cell specific markers MECA-32 and Ly6G 1A8 respectively displayed diminished alveolar changes related to COPD progression. Meijer et al. also implicated the importance of neutrophil involvement in COPD [31]; interestingly Btk inhibition may moderate neutrophil activity regarding excessive inflammation in the lungs. Both Btk and MMP-9 appear to be attractive targets for alleviation of COPD progression.

One limitation of our current research design involved using a pharmacological inhibitor of Btk which was capable of inhibiting Btk in cell types other than neutrophils. However, silencing Btk specifically in neutrophils is a viable route for future experiments; a method utilizing cell-specific siRNA targeting Btk in neutrophils has been previously established in our laboratory [12]. Further studies with a cell-specific siRNA for Btk may be ideal for targeting desired cell types within the lungs. Furthermore, our study design employed intravenous injection of both the Btk inhibitor and siRNA conjugates. Although this method is known to allow treatments such as ours to enter the lungs [32], an intranasal approach may potentially improve lung specific cell targeting, and therefore be a preferred approach for future studies involving siRNA-based treatments. This study is further limited by the lack of non-specific (scrambled) siRNA control treatment groups. However, in a previous publication from our lab [12] we demonstrated specificity of MMP-9 directed siRNA relative to control (scrambled) siRNA in vivo in neutrophils alongside BTK directed siRNA, additionally the Btk directed siRNA specificity was shown in vitro relative to control siRNA. In the previously employed short duration model fewer treatments were required and including these controls was both necessary for establishing specificity and economically feasible. For an experimental duration such as we used here with a premium modified siRNA product, unfortunately, addition of control siRNA groups was cost prohibitive. The siRNA used in both studies is a very high quality product which we have had great success with, therefore it was felt that addition of this control group would not alter the results obtained under the current design. Additionally, the current study observed silencing of MMP-9 exclusively in endothelial cells and neutrophils. Future studies regarding both Btk and MMP-9 in other cell types such as macrophages are of interest for our lab and perhaps others [33]. Irrespective of the limitations, our findings provide evidence that through Btk signaling MMP-9 may play a significant role in the development of lung inflammation and ultimately the progression of COPD.

## 4. Methods

### 4.1. Animal Studies

All studies involving animals were approved by the IACUC (Institutional Animal Care and Use Committee) at the University of Texas Health Science Center at Tyler (protocol 514, initial approval received 04/25/2012), and conform to National Institutes of Health guidelines. Age matched female Murine Pathogen Free ApoE^−/−^ mice were divided into four treatment groups consisting of either animals fed a high fat, high cholesterol Western Diet (WD) (D12079B, Research Diets Inc., New Brunswick, NJ, USA) with and without SHS exposure or animals fed standard rodent chow (PicoLab Rodent Diet 20, LabDiet, St. Louis, MO, USA) with and without SHS exposure. Mice were exposed to passive cigarette smoke using a whole-body smoke exposure system (TE-10B, Teague Enterprises, Woodland, CA, USA), as previously described [16]. Briefly, mice were exposed to a combination of 11% mainstream and 89% side stream smoke from 40 3R4F reference cigarettes twice daily, 5 days a week for up to 11 weeks. Control (nonsmoking) animals were exposed to ambient air only. Treatment animals were injected intravenously [tail vein injection] with either Bruton’s tyrosine kinase inhibitor (BTK Inh) PCI-32765 (Selleck Chemicals, Houston, TX, USA) or siRNA specific for MMP-9 (Invitrogen, Carlsbad, CA, USA) conjugated with F(ab’)_2_ fragments of anti-mouse neutrophil antibody (clone Ly-6G 1A8, Bio X Cell, West Lebanon, NH, USA) or anti-mouse endothelial cell antibody (clone MECA-32, Bio X Cell). F(ab’)_2_ fragments were generated with Pierce F(ab’)_2_ Preparation Kit (Thermo Fisher Scientific, Waltham, MA, USA) and conjugated to a siRNA carrier using T3-Max Conjugation Kits (Bioo Scientific, Austin, TX, USA). Treatments began after 7 weeks of WD/SHS exposure and lasted 2 or 4 weeks while continuing regular WD/SHS exposure. Control animals were exposed only to WD/SHS throughout the experiments. Five to six animals per treatment group and four to five animals per control group were used.

Following SHS exposure with/without treatment, mice were euthanized and the thoracic cavity was opened; blood was collected directly from the right ventricle. The renal vein was then cut and excess blood was flushed from the vasculature through the heart with cold PBS. The largest lung lobe was fixed for histology using ExCell Plus (American MasterTech, Lodi, CA, USA) and the remaining lobes were homogenized for further analysis.

### 4.2. Histochemical Evaluation of Emphysemic Changes

For histochemical analysis, fixed lungs were embedded in paraffin and sectioned for staining. Tissue sections were stained with Hart’s Elastin, Picro-Sirius Red (PSR), Hematoxylin and Eosin.

Differences in hue of PSR red stained airways were evaluated using ImageJ software. For airway analysis images were converted to RGB format, then the airway was selected as the region of interest (ROI) and mean intensities of only the red components were measured to exclude background and thin (green) fibers. Results were expressed as red intensity for each ROI. Differences in lung vasculature collagen content were measured in ImageJ by converting color images to gray-scale, selecting vasculature as the ROI, and measuring mean intensities. MLI was calculated to quantify airspace enlargement according to a previously described method [21] using Hematoxylin and Eosin stained lung tissue sections. Hart’s Elastin stained lung sections were employed to analyze a destruction of alveolar walls (breaks in elastic fibers) [17,18,19,20].

### 4.3. Western Blotting

Lung homogenates were normalized for equal protein concentration based on Bradford assay results, denatured in reducing Laemmle sample buffer, and then loaded into SDS-PAGE gel. Separated proteins were transferred to a polyvinylidene difluoride membrane (Pall Corp., Port Washington, NY, USA). The membrane was blocked and incubated with anti-MMP-9 antibody (C-20), anti-Actin antibody (I-19), or anti-cyclophilin D antibody (C-14) (Santa Cruz Biotechnology, Dallas, TX, USA) followed by HRP conjugated secondary antibodies (Jackson ImmunoResearch, West Grove, PA, USA). Bound antibodies were detected using enhanced chemiluminescence reagents (Bio-Rad Clarity ECL, Hercules, CA, USA). Finally, the membrane was exposed to X-ray film (HXR0810, Hawkins X-Ray Supply, Oneonta, AL, USA).

### 4.4. Statistics

Results are expressed as the mean ± STD. Differences between multiple groups were evaluated by one way ANOVA or the non-parametric Kruskal-Wallis ANOVA on ranks as dictated by variance/normality followed by post hoc analysis with Fisher’s least significant difference test or Dunn’s multiple comparison test respectively. In cases where Dunn’s test was unable to yield a direct evaluation for significance comparisons between groups of interest were performed using the nonparametric Mann-Whitney test with Bonferroni correction for multiple comparisons. Differences between paired groups were evaluated with Student’s *t*-test or the non-parametric Mann-Whitney rank sum test as dictated by variance/normality. The specific analyses used are indicated in individual figure legends. All statistics were performed using SIGMAPLOT 11 (Systat Software Inc., San Jose, CA, USA). Significance was defined as *p* < 0.05.

## 5. Conclusions

Cell specific targeting of Bruton’s tyrosine kinase (Btk) and/or matrix metalloproteinase-9 (MMP-9) may have potential as treatments for COPD in patients. Inhibition of Btk showed promising protective effects in the lungs of our animal model, WD fed, SHS exposed ApoE^−/−^ mice. Similarly, silencing MMP-9 in endothelial cells or neutrophils considerably diminished the progression of COPD characteristics in this animal model. Focusing prospective treatment options around disrupting the Btk pathway may prove to be ideal pursuits for future clinical research in COPD.

## Supplementary Materials

Supplementary materials can be found at http://www.mdpi.com/1422-0067/19/2/343/s1.

## Abbreviations

ApoE^−/−^	apolipoprotein E-deficient
Btk	Bruton’s tyrosine kinase
Btk Inh.	Bruton’s tyrosine kinase inhibitor PCI-32765 (Ibrutinib)
COPD	Chronic obstructive pulmonary disease
Ly6G	Monoclonal antibody (clone 1A8) against Lymphocyte Antigen 6 Complex Locus G, a specific marker of mouse neutrophils
MECA-32	Monoclonal antibody (clone MECA-32) against mouse pan-endothelial cell antigen, a specific marker of mouse endothelial cells
MMPs	matrix metalloproteinases
MMP-9	matrix metalloproteinase-9
PSR	picro-sirius red
SHS	second hand smoke
WD	Western Diet
