# Supplementary material for: Disrupting the Btk Pathway Suppresses COPD-Like Lung Alterations in Atherosclerosis Prone ApoE−/− Mice Following Regular Exposure to Cigarette Smoke
Source: Int J Mol Sci. 2018 Jan 24;19(2):343. doi: 10.3390/ijms19020343 (PMC5855565; doi:10.3390/ijms19020343)
Supplement: Supplementary file 1 [file ijms-19-00343-s001.pdf]

Supplementary

Disrupting the Btk Pathway Suppresses COPD-Like Lung Alterations in Atherosclerosis Prone ApoE$^{-/-}$ Mice Following Regular Exposure to Cigarette Smoke

Jon M. Florence [1], Agnieszka Krupa [1,2], Laela M. Booshehri [1], Adrian L. Gajewski [1,2] and Anna K. Kurdowska [1,*]

1 Department of Cellular and Molecular Biology, University of Texas Health Science Center, Tyler, TX 75708, USA; jon.florence@uthct.edu (J.M.F); agakrupa@yahoo.com (A.K.); laela.booshehri@gmail.com (L.M.B.); gajewski@biol.uni.lodz.pl (A.L.G.)

2 Laboratory of Gastroimmunology, Department of Immunology and Infectious Biology, Faculty of Biology and Environmental Protection, University of Lodz, 90-136 Lodz, Poland

* Correspondence: anna.kurdowska@uthct.edu; Tel.: +1-903-877-7738

Supplement Methods

Confocal microscopy

Lung tissue sections were evaluated for the expression of Btk and MMP-9 using Perkin Elmer (Waltham, MA, USA) UltraVIEW LCI confocal imaging system with a Nikon TE2000-S fluorescence microscope and PlanApo360 immersion oil objective (numerical aperture 1.4) at room temperature. UltraVIEW Imaging Suite software (version 5.5.0.4) was used for image processing. Briefly, the sections were incubated with primary antibodies (anti-mouse Btk or anti-mouse MMP-9, Santa Cruz Biotechnology, Dallas, TX, USA or anti-mouse MMP-9 Millipore, Burlington, MA, USA) followed by secondary antibodies conjugated with fluorescent dyes (Invitrogen, Carlsbad CA, USA). For some staining Hoechst 33342 (Calbiochem, San Diego, CA, USA) was added to visualize nuclei.

Gelatin Zymography and Densitometry

MMP-9 enzymatic activity was assayed in lung homogenates by gel electrophoresis gelatin zymography as previously described (16). Lung homogenates were normalized for equal protein concentration based on Bradford assay results, denatured in non-reducing Laemmli sample buffer, and then separated in an 8% polyacrylamide gel containing 0.05% gelatin under constant voltage. After electrophoresis gels were removed from cassettes and enzymes were allowed to re-fold in Novex Zymogram Renaturing Buffer (Life Technologies; Grand Island, NY, USA) at room temperature for 90 min, replacing buffer every 30 minutes. Gels were then rinsed and incubated at 37°C in Novex Zymogram Developing Buffer (Life Technologies; Thermo Fisher Scientific; Waltham, MA, USA) containing 0.02% sodium azide for 90 hours. Following incubation gels were soaked 45 minutes in 20% trichloroacetic acid, then stained with Brilliant Blue R250 (Sigma Aldrich; St. Louis, MO, USA) and de-stained until zones of digestion were visible as clear bands against a blue background. The destained gel was imaged in grayscale with a ChemiDoc Imaging System (Bio-Rad, Hercules, CA, USA), and the image was inverted to give dark bands on a light background. ImageJ software was used to analyze a band at approximately 90-100kDa (corresponding to MW of MMP-9) and calculate densitometry values. For each group, the average ± SEM value was graphed.

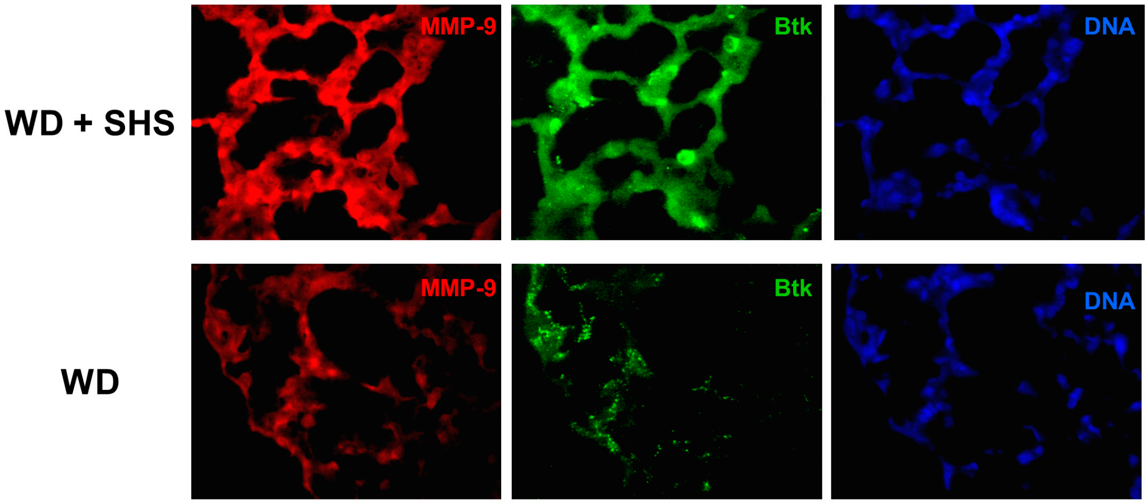

Figure S1: Increased Levels of MMP-9 and Btk in Lung Tissue of SHS exposed Mice. Fluorescent immunohistochemical staining was used to show increases in Btk (green) and MMP-9 (red) in lung tissue of WD fed, SHS exposed mice relative to mice fed WD alone. DNA counterstain (blue) was also included.

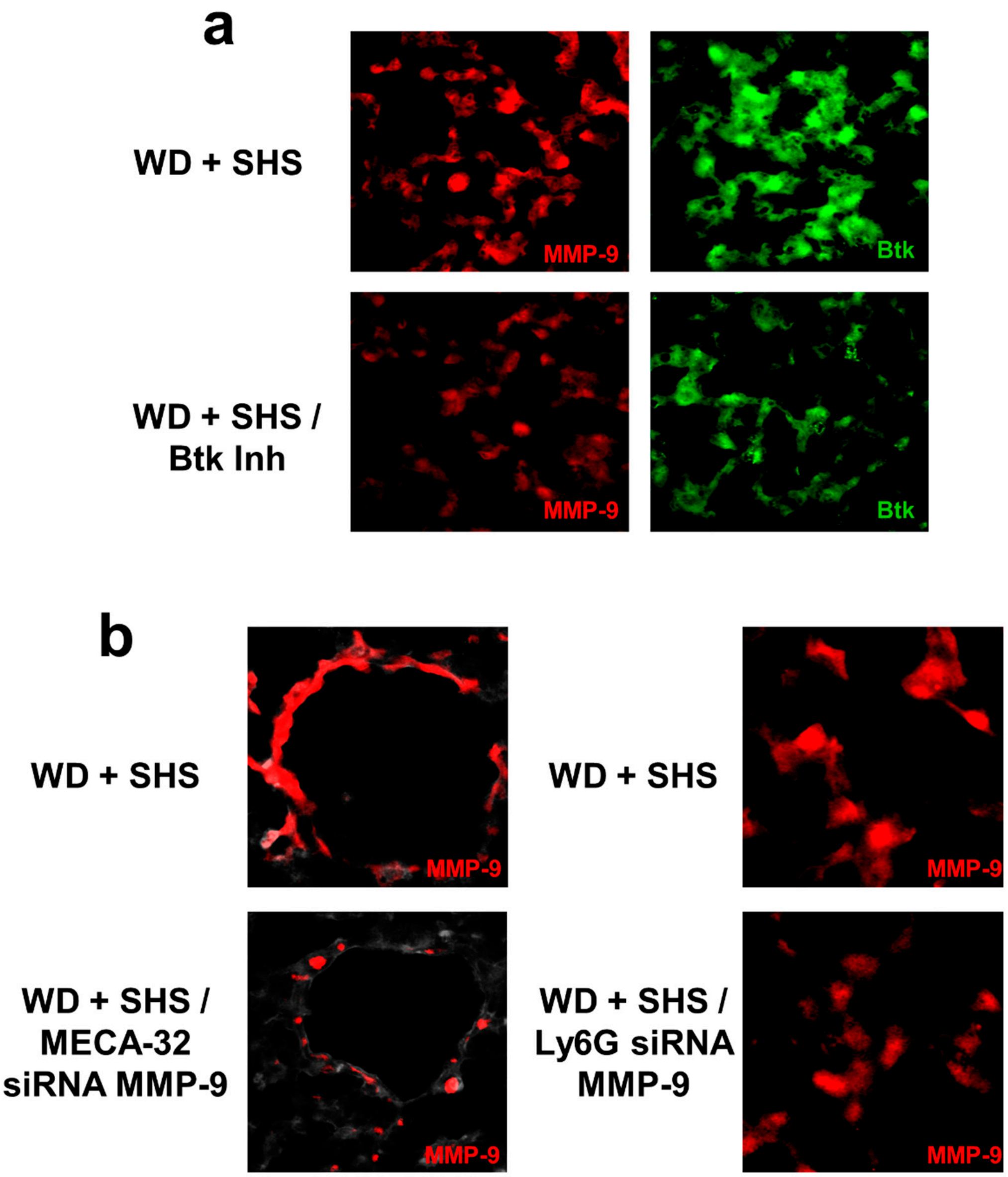

Figure S2: Decreased Levels of Btk and/or MMP-9 in Lung Tissue from Treated Mice. Fluorescent immunohistochemical staining was used to show decreases in (a) Btk (green) and MMP-9 (red) in lung tissue of Btk Inh treated, WD + SHS exposed mice relative to WD + SHS control mice, and (b) MMP-9 (red) in lung tissue of MECA-32 siRNA MMP-9 treated WD + SHS exposed (left) and Ly6G siRNA MMP-9 treated WD + SHS exposed (right) mice relative to corresponding WD + SHS exposed controls.

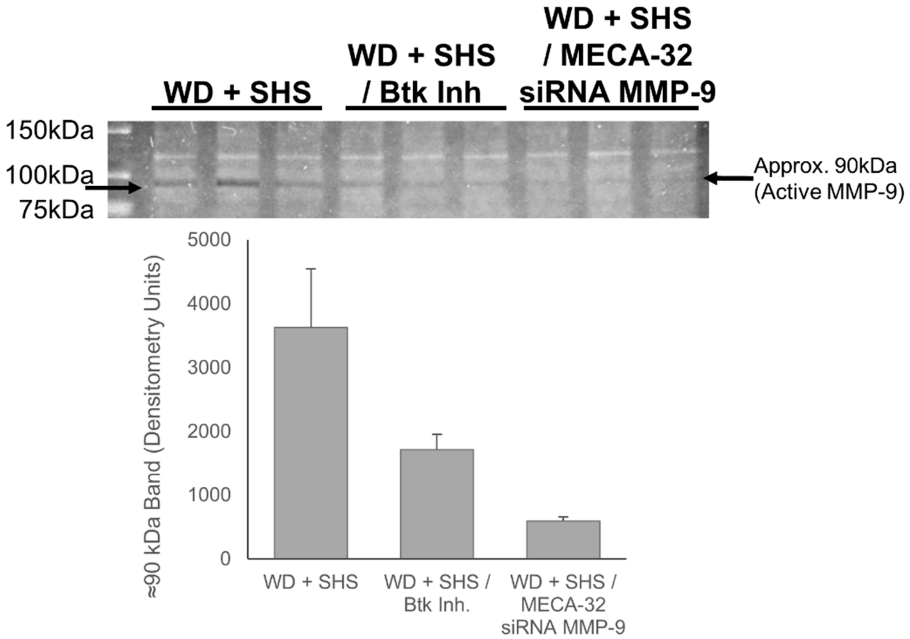

Figure S3: Gelatin Zymography of Reduced MMP-9 Activity in Lung Homogenates from Btk Inh and MECA-32 siRNA MMP-9 Treated Mice. Gel electrophoresis gelatin zymography was used to show reduced gelatinolytic activity in the 90-100 kDa range corresponding to MMP-9 activity present in lung homogenates. Corresponding densitometry values for each group are graphed as well, average ± SEM for 3 mice per group shown.
